# Glycemic Control and Associated Factors Among Children With Type 1 Diabetes Mellitus at the Pediatric Diabetic Clinic of Yekatit 12 Hospital Medical College, Addis Ababa, Ethiopia

**DOI:** 10.1155/jdr/7285054

**Published:** 2026-11-06

**Authors:** Mekashaw Wudu, Senbeto Gehano, Minase Shenkute, Alemayehu Tsegaye

**Affiliations:** ^1^ Department of Pediatrics and Child Health School of Medicine Yekatit 12 Hospital Medical College Addis Ababa Ethiopia; ^2^ Department of Pediatrics and Child Health School of Medicine Institute of Health Sciences Dilla University Dilla Ethiopia du.edu.et; ^3^ Department of Anesthesiology and Critical Care Medicine School of Medicine Institute of Health Sciences Dilla University Dilla Ethiopia du.edu.et

**Keywords:** Ethiopia, glycemic control, pediatrics, Type 1 DM

## Abstract

**Introduction:**

Type 1 DM is the most common endocrine‐metabolic disorder of childhood, and poor glycemic control increases the risk of long‐term complications and mortality. Despite this burden, evidence on glycemic control and its associated factors among children with Type 1 DM in Ethiopia remains limited.

**Objective:**

The aim of the study is to assess glycemic control and its associated factors among children with T1DM attending the pediatric diabetes clinic at Yekatit 12 Hospital Medical College, Addis Ababa, Ethiopia.

**Methods:**

A cross‐sectional study was conducted among 181 children from September 1 to November 30, 2023. Data were collected using a structured questionnaire and analyzed using SPSS Version 25. Variables with *p* < 0.25 in binary logistic regression were entered into multivariable logistic regression, with adjusted odds ratios (AOR), 95% confidence intervals (CI), and *p* < 0.05 used to declare significance.

**Results:**

Among the 181 participants, 59 (32.6%) had good glycemic control, whereas 122 (67.4%) had poor glycemic control. On multivariable logistic regression, caregiver illiteracy (AOR 5.36, 95% CI: 1.41–20.47, *p* = 0.014) and presence of lipodystrophy at the injection site (AOR 11.29, 95% CI: 3.03–41.99, *p* = 0.003) were independently associated with poor glycemic control.

**Conclusion:**

Poor glycemic control is highly prevalent among children with Type 1 DM at Yekatit 12 Hospital Medical College. Caregiver illiteracy and lipodystrophy at the injection site were associated with poor glycemic control. Multicenter studies are recommended to validate these findings in similar resource‐limited settings.

## 1. Introduction

Poor glycemic control in children with Type 1 diabetes mellitus (T1DM) is a major public health concern because it is associated with increased healthcare costs, reduced quality of life, and microvascular and macrovascular complications, including nephropathy, neuropathy, retinopathy, and cardiovascular disease [1]. Achieving glycemic targets is the primary therapeutic goal to prevent these complications [2], yet most patients fail to reach them [3], and only 21% of adolescents with T1DM achieve recommended targets according to the American Diabetes Association [1]. In sub‐Saharan Africa, outcomes among children with T1DM remain poor because of delayed diagnosis, limited access to healthcare resources, and inadequate diabetes education [4]. Furthermore, T1DM has received limited research attention, partly because confirmatory testing can be costly [5].

In Ethiopia, suboptimal glycemic control has been linked to inadequate training of healthcare workers, limited access to essential medications and technologies, poor awareness, and inadequate documentation systems [3]. Although children often receive donated insulin and glucose‐monitoring supplies, families frequently incur substantial out‐of‐pocket costs during shortages [6]. Poor glycemic control was reported in 39.3% of children in northwestern Ethiopia [3] and 83.6% in southern Ethiopia. In the latter study, lipodystrophy and inability to afford insulin were significantly associated with poor glycemic control [6]. Studies from Tanzania and Kenya also reported poor control in most participants [7, 8].

Evidence from Yekatit 12 Hospital Medical College, a major referral center in Addis Ababa, remains limited. This study is aimed at assessing glycemic control and its associated factors among children with T1DM attending the pediatric diabetes clinic at Yekatit 12 Hospital Medical College in Addis Ababa, Ethiopia.

## 2. Materials and Methods

### 2.1. Study Area

The study was conducted at Yekatit 12 Hospital Medical College, a major public teaching hospital in Addis Ababa, Ethiopia. The hospital provides specialized and referral healthcare services to residents of Addis Ababa and patients referred from other regions of Ethiopia. The hospital provides a wide range of preventive, diagnostic, curative, and rehabilitative services through its various departments and specialty clinics. The pediatric diabetes follow‐up clinic provides ongoing care and management for children and adolescents with diabetes mellitus.

### 2.2. Study Design and Period

A hospital‐based cross‐sectional study was conducted from September 1 to November 30, 2023.

### 2.3. Population

#### 2.3.1. Source Population

The source population is all children with T1DM receiving follow‐up care at Yekatit 12 Hospital Medical College.

#### 2.3.2. Study Population

The study population is all children with T1DM receiving follow‐up care at Yekatit 12 Hospital Medical College during the study period, from September 1 to November 30, 2023.

### 2.4. Inclusion and Exclusion Criteria

#### 2.4.1. Inclusion Criteria

The inclusion criteria are as follows:

• Children aged 14 years or younger who had been diagnosed with T1DM, were receiving treatment, and had attended the diabetes clinic for at least 6 months were included.

#### 2.4.2. Exclusion Criteria

The exclusion criteria are as follows:

• Those children who are on treatment for less than 6 months.
• Children who were critically ill.
• Children who did not have a recent HbA1c measurement.

### 2.5. Sample Size Determination and Sampling Technique

#### 2.5.1. Sample Size Determination

The sample size was determined using the single population proportion formula, assuming a prevalence of poor glycemic control of 83.6% based on a previous study conducted in southern Ethiopia [6]. A 95% confidence level and a 5% margin of error were used in the calculation.

n=zα/22p1−Pd2=1.9620.83610.836−0.052=211,

where *n* is the required sample size, *Z* *α*/2 is the critical value for normal distribution at 95% confidence level, *p* is the assumed prevalence of poor glycemic control, *d* = 0.05 (5% margin of error).

After accounting for a 10% nonresponse rate, the calculated sample size was 233. Because the estimated number of children attending the clinic was comparable with the required sample size, all eligible children were considered for inclusion.

#### 2.5.2. Sampling Procedure and Sampling Technique

An estimated 260 children in the pediatric age group were followed up at the Diabetic Clinic of Yekatit 12 Hospital Medical College. A total population sampling technique was used, in which all eligible children were included in the study. However, 79 children (30.4%) were excluded from the study: 58 (73.4%) did not have a recent HbA1c determination (within the last 3 months), and 21 (26.6%) were absent from the clinic during the data collection period. A lower sample than planned may reduce statistical precision and power.

### 2.6. Data Collection Methods

#### 2.6.1. Data Collection Instruments

For this study, a structured questionnaire was used. The questionnaire was prepared in English and then translated into Amharic. It included sociodemographic data, diabetes history, insulin regimen and dosage, and diabetes‐related practices. This tool was adopted from several previously published studies, with some modifications.

#### 2.6.2. Data Collectors

Data were collected by two general practitioners.

#### 2.6.3. Procedure of Data Collection

Data were collected using a structured, paper‐based questionnaire administered during caregiver interviews, direct examinations, and reviews of medical records. Sociodemographic characteristics, diabetes history, and diabetes‐related practices were obtained by interview. Anthropometric measurements were taken at the clinic by triage staff before the child was seen by the physician in the follow‐up room. The most recent HbA1c value was extracted from the electronic medical record. The data collection was carried out over 3 months from September 1 to November 30, 2023. The data quality was checked for clarity and completeness.

#### 2.6.4. Assessment of Lipodystrophy

Injection‐site examination was performed by the data collectors at the clinic visit. Lipodystrophy was recorded as present if lipohypertrophy (a visible or palpable swelling or thickened, rubbery area of subcutaneous tissue) or lipoatrophy (a visible depression or loss of subcutaneous fat) was found at any site and absent otherwise.

### 2.7. Study Variables

#### 2.7.1. Dependent Variable

The dependent variable is the level of glycemic control.

#### 2.7.2. Independent Variable

The independent variables are as follows:

• Sociodemographic factors: age, sex, caregiver educational level, caregiver marital status, occupation, place of residence, family size, and household income.
• Clinical and medication‐related factors: age at diagnosis, duration of diabetes, duration of treatment, anthropometric measurements, insulin type and dose, comorbidities, medication use, insulin storage conditions, and injection‐site findings.
• Nutritional factors: The number of meals per day, meal content.
• Health facility factors: Frequency of clinic visits, the distance of health facility, counseling by a health professional, and presence of health insurance.

### 2.8. Operational Definitions

#### 2.8.1. Glycemic Control Was Assessed Using HbA1c Levels

• Good glycemic control—HbA1c is less than 7.5% [9].
• Poor glycemic control—if HbA1c is more than or equal to 7.5% [9].
• Recent HbA1c—HbA1c determination in the last 3 months.

#### 2.8.2. Anthropometry Parameters—Length, Weight

• Length measured using a length board in children younger than 2 years, whereas standing height was measured using a stadiometer in older children.
• Weight—measured using a weighing scale.

1. Weight for age, between 3SD and −2SD—normal and below −3SD—malnourished
2. Length/height for age, between 3SD and −2SD—normal and below −3SD—malnourished.
3. Weight for length/height, between 3SD and −2SD—normal and below −3SD—malnourished.
4. BMI for age, between 3SD and −2SD—normal and below −3SD—malnourished

Comorbidities are defined as coexisting conditions, including cardiac disease, hypertension, and HIV infection.

### 2.9. Data Quality Control

After the questionnaire preparation and before actual data collection, the pretest was conducted among 10 participants at Yekatit 12 Hospital Medical College. The data were checked daily for completeness and consistency before processing and analysis. Both data collectors received half a day of training on the study objectives and data collection procedures.

### 2.10. Data Processing and Analysis

The data were checked for completeness, coded, and entered into SPSS Version 25 for analysis. Descriptive statistics, including frequencies, proportions, means, and standard deviations, were used to summarize the study variables. Glycemic control was classified as good when HbA1c was < 7.5% and poor when HbA1c was ≥ 7.5% [9].

Binary logistic regression analysis was performed to identify factors associated with poor glycemic control. In the bivariable analysis, crude odds ratios (COR) with 95% CI were calculated. Variables with a *p* value < 0.25 in the bivariable analysis were considered candidates for multivariable logistic regression. Multicollinearity among candidate independent variables was assessed using the variance inflation factor (VIF) and all VIF values were < 2, indicating no evidence of problematic multicollinearity. Variables meeting the predefined screening criterion were then entered into the multivariable logistic regression model.

The final multivariable logistic regression model was used to identify factors independently associated with poor glycemic control. Model goodness‐of‐fit was assessed using the Hosmer–Lemeshow test, which indicated adequate model fit (*p* = 0.456). Adjusted odds ratios (AORs) with 95% CIs were reported, and a *p* value < 0.05 was considered statistically significant.

## 3. Results

### 3.1. Sociodemographic Characteristics of Children With Type 1 DM

Among the 181 participants, 31 (17.1%) were aged 0–4 years, 88 (48.6%) were aged 5–9 years, and 62 (34.3%) were aged 10–14 years. Of the participants, 92 (50.8%) were male and 89 (49.2%) were female.

Regarding educational status, 78 children (43.1%) were preschool‐aged and not yet eligible to attend school, 92 (50.8%) were in elementary school, and 11 (6.1%) were in high school (Table 1).

**Table 1:** Sociodemographic characteristics of participants with Type 1 DM in Yekatit 12 Hospital Medical College on diabetic clinic, November 2023 (*N* = 181).

Variable	Category	Frequency	Percentage
Age	0–5	31	17.1
	5–10	88	48.6
	10–14	62	34.3

Sex	Male	92	50.8
	Female	89	49.2

Educational status	Preschool	78	43.1
	Elementary	92	50.8
	High school	11	6.1

### 3.2. Sociodemographic Characteristics of Primary Caregivers

Most participants were primarily cared for by their mothers (129, 71.3%), followed by their fathers (25, 13.8%), siblings (23, 12.7%), and other relatives (4, 2.2%).

Regarding caregiver educational status, 32 (17.7%) were classified as illiterate and 149 (82.3%) as literate. Marital status of the primary caregivers: 28 (15.5%) of them were single, 136 (75.1%) of them married, 7 (3.9%) were separated, and 10 (5.5%) divorced.

Regarding caregiver occupation, 19 (10.5%) were government employees, 45 (24.9%) worked in private institutions, 77 (42.5%) were homemakers, 10 (5.5%) were merchants, and 30 (16.6%) had other occupations, including unemployment, daily labor, and other informal work.

Regarding place of residence, six participants (3.3%) lived in rural areas, whereas 175 (96.7%) lived in urban areas. For family size, 91 households (50.3%) had 2–4 members, 89 (49.2%) had 5–10 members, and 1 (0.6%) had more than 10 members.

According to the income categories used in the study, 97 participants (53.6%) were in the low‐income category, 76 (42.0%) were in the lower middle‐income category, and 8 (4.4%) were in the upper middle‐income category. No participants were classified as high‐income (Table 2).

**Table 2:** Sociodemographic characteristics of primary caregivers of participants with type 1 DM in Yekatit 12 Hospital Medical College on diabetic clinic, November 2023 (*N* = 181).

Variable	Category	Frequency	Percentage
Primary caregiver	Mother	129	71.3
	Father	25	13.8
	Sister/brother	23	12.7
	Other	4	2.2

Sex	Male	20	11.0
	Female	161	89.0

Educational level	Illiterate	32	17.7
	Literate	149	83.3

Marital status	Single	28	15.5
	Married	136	75.1
	Separated	7	3.9
	Divorced	10	5.5

Occupation	Government employee	19	10.5
	Private institution	45	24.9
	Housewife	77	42.5
	Merchant	10	5.5
	Others	30	16.6

Residence	Rural	6	3.3
	Urban	175	96.7

Family size	2–4	91	50.3
	5–10	89	49.2
	> 10	1	0.6

Monthly income	Low (< 5154)	97	53.6
	Lower middle (5155–20215)	76	42.0
	Upper middle (20,216–62,724)	8	4.4

### 3.3. Nutritional Characteristic of Participants

Regarding daily meal frequency, 45 participants (24.9%) consumed three meals and two or three snacks, 95 (52.5%) consumed three meals and one snack, and 41 (22.7%) consumed three meals without snacks. From the respondents 66(36.5%) of them consume foods which are forbidden for diabetes and from these most foods are 5 (2.8%) sugary foods like tea, 55 (30.4%) pasta and macaroni, packed foods like juice, mirinda (15 (8.3%), foods sugar added 14 (7.7%).

Children younger than 5 years were assessed using weight‐for‐age, weight‐for‐height, height‐for‐age, and MUAC. Children aged 5 years and older were assessed using BMI (Table 3).

**Table 3:** Nutritional characteristics of participants with Type 1 DM in Yekatit 12 Hospital Medical College on diabetic clinic, November 2023 (*N* = 181).

Variable	Category	Frequency	Percentage
Meals/day	3 meals + 2–3 snacks	45	24.9
	3 meals + 1 snack	95	52.5
	3 meals only	41	22.7

Forbidden food	Yes	66	36.5
	No	115	63.5

WFA	Moderate underweight	3	1.7
	Normal	73	40.3
	Not assessed	105	58.0

WFH	Normal	73	40.3
	Not assessed	108	59.7

MUAC	11–12.5 cm	6	3.3
	≥ 12.5 cm	68	37.6
	Not assessed	107	59.1

BMI	Normal	129	71.3
	Not assessed	52	28.7

### 3.4. Clinical Characteristics of Participants

Regarding age at diagnosis, 85 participants (47.0%) were diagnosed before age 3 years, 44 (24.3%) were diagnosed at ages 4–6 years, and 52 (28.7%) were diagnosed at age 7 years or older.

Most participants (128, 70.7%) had lived with diabetes for 1–5 years, whereas 29 (16.0%) had been diagnosed less than 1 year earlier and 24 (13.3%) had lived with diabetes for more than 5 years. All of the respondents start insulin immediately after diagnosis.

All participants used a twice‐daily premixed insulin regimen containing regular insulin and NPH insulin. Regarding total daily insulin dose, 91 participants (50.3%) received less than 0.8 IU/kg/day, 76 (42.0%) received 0.8–1.2 IU/kg/day, and 14 (7.7%) received more than 1.2 IU/kg/day.

Lipodystrophy was identified at the insulin injection site in 29.8% of participants.

Regarding insulin storage, 168 participants (92.8%) stored insulin in a refrigerator, whereas 13 (7.2%) used a pot‐based storage method. Insulin was administered by the children themselves in 39 cases (21.5%), by caregivers in 122 cases (67.4%), and by children with caregiver assistance in 20 cases (11.0%).

During the preceding week, 175 participants (96.7%) reported missing no insulin doses, whereas 6 (3.3%) reported missing one dose. All of the respondents have glucometers. Insulin doses given by a caregiver; 131 of them given all the doses by the caregivers and 01 injection for six (3.3%) of them and 44 (24.3%) of them by themselves.

During interviews in the last 24 h, all injections were supervised by caregivers for 134 participants (74%), one injection was supervised for 11 participants (6.1%), and injections were self‐administered by 36 participants (19.9%).

For 122 participants (67.4%), caregivers set up the glucose meter, performed the finger prick, or supervised the procedure. For 46 participants (25.4%), caregivers reminded the child to record or report blood glucose levels. For the remaining 13 participants (7.2%), caregivers did not participate in glucose monitoring.

Of the total 181 respondents, those with good glycemic control based on HbA1c accounted for 59 (32.6%), whereas the remaining 122 (67.4%) had poor glycemic control with an HbA1c level ≥ 7.5% (Figure 1).

**Figure 1:**
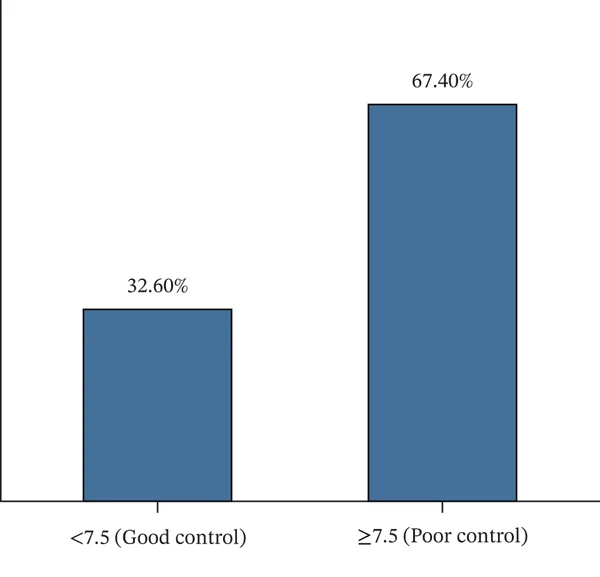
Glycemic control of participants with Type 1 DM in Yekatit 12 Hospital Medical College on diabetic clinic, November 2023 (*N* = 181).

All participants received physician counseling at the time of diagnosis on the importance of insulin therapy, insulin administration, glucometer use, diabetes complications, adherence to insulin doses and clinic appointments, dietary management, and the management of hypoglycemia.

Regarding follow‐up frequency, 29 participants (16.0%) attended monthly or more frequently, 92 (50.8%) attended every 2 months, and 60 (33.1%) attended every 3 months. Sixty‐three participants (34.8%) reported problems during follow‐up. The reported problems included long waiting times to see a physician (56 participants, 30.9%) and administrative difficulties related to medical registration (20 participants, 11.0%).

From the total respondents 96 (53%) of them have health insurance and the remaining have not. (Figure 2).

**Figure 2:**
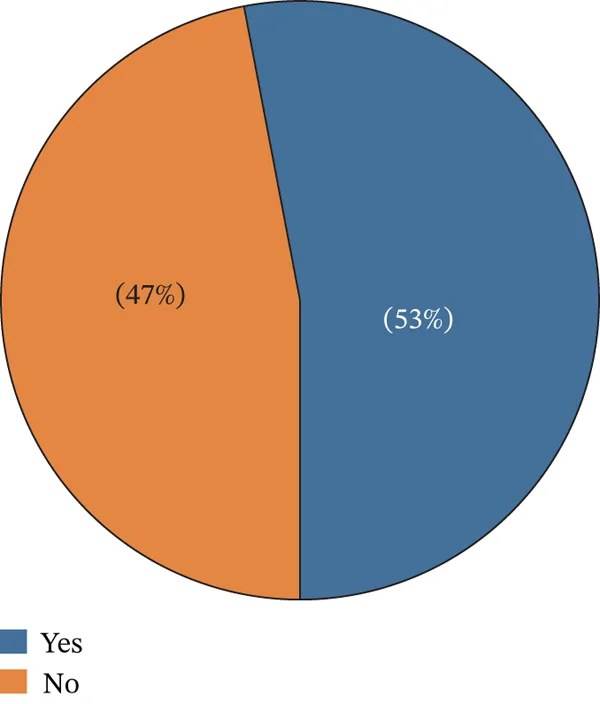
Percentage of the participants with Type 1 DM who have health insurance in Yekatit 12 Hospital Medical College on diabetic clinic, November 2023 (*N* = 181).

Regarding health system‐related characteristics, most participants lived within 10 km of the hospital (78.5%) with a travel time under 1 h (82.3%). Follow‐up visits were scheduled monthly for 16.0%, every 2 months for 50.8%, and every 3 months or more for 33.1% of participants. Nearly one‐third (34.8%) reported problems during follow‐up, predominantly long waiting times (30.9%). Health insurance coverage was reported by 53.0% of participants.

### 3.5. Factors Associated With Poor Glycemic Control

In the bivariable analysis, children aged 10–14 years, female caregivers, caregivers employed in private institutions and housewives, caregiver illiteracy, children receiving three meals plus one snack per day, lipodystrophy at the insulin injection site, diabetes duration of less than 1 year, a total daily insulin dose of 0.8–1.2 IU/kg/day, and caregivers who reminded children to monitor their blood glucose only were associated with poor glycemic control (Table 4). Variables with *p* values below 0.25 in the bivariable analysis were entered into the multivariable logistic regression model. After adjustment, caregiver illiteracy (AOR 5.36, 95% CI: 1.41–20.47, *p* = 0.014) and lipodystrophy at the injection site (AOR 11.29, 95% CI: 3.03–41.99, *p* = 0.003) remained independently associated with higher odds of poor glycemic control (Table 5).

**Table 4:** Bivariable logistic regression analysis of factors associated with poor glycemic control of participants with Type 1 DM in Yekatit 12 Hospital Medical College on diabetic clinic, November 2023 (*N* = 181).

Variable	Category	Good, *n*	Poor, *n*	COR (95% CI)	*p*
Child age (years)	0–5	12	19	1	
	5–10	33	55	1.05 (0.45–2.44)	0.905
	10–14	14	48	2.16 (0.84–5.52)	0.106

Caregiver sex	Female	55	106	0.48 (0.15–1.51)	0.211
	Male	4	16	1	

Caregiver occupation	Government employee	3	16	1	
	Private institution	21	24	0.21 (0.05–0.84)	0.027
	Housewife	26	51	0.37 (0.10–1.38)	0.138
	Merchant	2	8	0.75 (0.10–5.43)	0.776
	Others	7	23	0.62 (0.14–2.75)	0.526

Caregiver education	Literate	55	94	1	
	Illiterate	4	28	4.10 (1.36–12.29)	0.012

Meals per day	3 meals only	17	24	1	
	3 meals + 1 snack	28	67	1.69 (0.79–3.63)	0.175
	3 meals + 2–3 snacks	14	31	1.57 (0.65–3.80)	0.319

Lipodystrophy at injection site	No	52	75	1	
	Yes	7	47	4.655 (1.952–11.103)	0.001

Age at diagnosis (years)	< 3	31	54	0.64 (0.29–1.41)	0.250
	3–6	14	30	0.79 (0.32–1.94)	0.599
	≥ 7	14	38	1	

Duration of diabetes	< 1 year	13	16	0.41 (0.13–1.33)	0.138
	1–5 years	40	88	0.73 (0.27–1.99	0.542
	≥ 5 years	6	18	1	

Total insulin dose (IU/kg/day)	< 0.8	26	65	0.68 (0.18–2.64)	0.580
	0.8–1.2	30	46	0.42 (0.11–1.62)	0.208
	≥ 1.2	3	11	1	

Caregiver role in glucose monitoring	Sets up meter/does finger prick	45	77	1	
	Reminds child only	9	37	2.40 (1.06–5.43)	0.035
	No participation	5	8	0.94 (0.29–3.03)	0.911

*Note:* Poor glycemic control was Coded 1 and good control Coded 0.

**Table 5:** Multivariable logistic regression analysis of factors associated with poor glycemic control of participants with Type 1 DM in Yekatit 12 Hospital Medical College on diabetic clinic, November 2023 (*N* = 181).

Variable	Glycemic control		AOR at 95% CI	*p*
	Good	Poor		
Caregiver education				
Illiterate	4	28	5.362 (1.405, 20.465)	0.014
Literate	55	94	1	

Lipodystrophy at injection site				
Yes	7	47	11.285 (3.033, 41.993)	0.003
No	52	75	1	

*Note:* Poor glycemic control was Coded 1 and good control Coded 0.

## 4. Discussion

This study assessed the prevalence of poor glycemic control and its associated factors among children with T1DM attending Yekatit 12 Hospital Medical College. Overall, 67.4% of participants had poor glycemic control.

The prevalence of poor glycemic control in our study was comparable with that reported in studies from sub‐Saharan Africa (71.9%), Cameroon (72%), Kenya (67.4%), and Harar, eastern Ethiopia (76%) [1, 4, 8, 10]. The prevalence in our study was lower than that reported in Hawassa (83.6%), China (80%), Saudi Arabia (75%), and Tanzania (97.3%) [6, 11–13], but higher than that reported in northwestern Ethiopia (39.3%) and Europe (34%) [3, 14].

Direct comparison between these studies is limited by differences in the HbA1c cutoff used to define poor control (≥ 9% in the Cameroon and China studies [4, 11], ≥ 8% in the Kenya study [8], and ≥ 7% in the northwestern Ethiopian study [3], compared with ≥ 7.5% in our study), in the age range of participants, and in the study setting (single hospital versus multicenter or registry‐based samples). The lower prevalence reported in the European cohort is consistent with the possibility that these settings have greater access to insulin analogs, continuous glucose monitoring, and insulin pumps [14], which were not available to the children in our clinic. We did not measure these factors, so this remains a possible explanation that our data cannot test.

Previous studies have identified several factors associated with glycemic control in children, including age, sex, body mass index, socioeconomic status, caregiver education, diabetes duration, insulin dose, and injection‐site lipodystrophy [10, 13, 15–17]. In the present study, caregiver illiteracy and lipodystrophy at the injection site remained independently associated with poor glycemic control after adjustment.

Caregiver illiteracy was associated with higher odds of poor glycemic control (AOR 5.36, 95% CI: 1.41–20.47, *p* = 0.014). A similar finding was reported in Tanzania, where children of caregivers with primary or higher education had better control than those whose caregivers had no formal education [7]. Because T1DM management often depends on caregivers who must understand insulin‐dosing instructions, monitor and record blood glucose levels, recognize hypoglycemia, and follow clinical advice, limited literacy may hinder effective day‐to‐day diabetes management [7]. The strength and consistency of this association make caregiver literacy an important marker for children who may need additional support, such as pictorial or verbal education materials and closer follow‐up.

Presence of lipodystrophic change at the injection site, which is associated with poor glycemic control, with 11times more likely to have poor glycemic control than these who have not *p* = 0.003, AOR 11,285(3.033, 41.993) Which is supported in study done southern Ethiopia (Hawassa) which was associated with fourfold increase in poor glycemic control [6]. Lipodystrophy is known to cause unpredictable insulin absorption, which may contribute to hyperglycemia. However, because this is a cross‐sectional study, the direction of the association cannot be established. Reverse causality is possible: children with poor control may receive higher or more frequent insulin doses, or may be more likely to repeat injections at familiar sites, which could promote lipodystrophy. Poor injection technique, needle reuse, and limited education, which are themselves linked to poor control, may also cause lipodystrophy and poor control together.

Although caregiver illiteracy and lipodystrophy at the injection site were independently associated with poor glycemic control, the corresponding confidence intervals were relatively wide. These wide intervals indicate limited precision of the estimated effect sizes and suggest that the magnitude of these associations should be interpreted cautiously. The imprecision may partly reflect the relatively small number of participants in some exposure categories, particularly caregivers who were illiterate and participants with lipodystrophy. Larger studies with adequate representation of these subgroups are needed to obtain more precise estimates of these associations.

## 5. Conclusion

More than two‐thirds of children with T1DM had poor glycemic control. Caregiver illiteracy and lipodystrophic changes at injection sites were significantly associated with poor glycemic control. As this was a single‐center study, multicenter research is recommended to validate these findings and inform diabetes care policy in similar resource‐limited settings.

## 6. Limitation

Of the estimated 260 children under follow‐up, 79 were excluded, mostly because they lacked a recent HbA1c measurement. If children without a recent HbA1c were less engaged with care or had irregular testing, selection bias is therefore possible, but its direction and magnitude are uncertain. The relatively wide confidence intervals observed for caregiver illiteracy and lipodystrophy indicate limited precision in the estimated effect sizes. This may partly reflect the relatively small number of participants in some exposure categories. Therefore, the magnitude of these associations should be interpreted cautiously, and larger studies are needed to provide more precise estimates. Data on insulin adherence, frequency of glucose monitoring, access to insulin, and household socioeconomic status were not collected, so these factors could not be adjusted for and residual confounding is possible. The adjusted associations of caregiver illiteracy and lipodystrophy with poor glycemic control should therefore be interpreted with caution, and future studies should collect these variables. Additionally, the single‐center and cross‐sectional nature of the study limits generalizability and does not allow causal inference.

**Nomenclature**
AOR: adjusted odds ratio
BMI: body mass index
CI: confidence interval
COR: crude odds ratio
DM: diabetes mellitus
HbA1c: glycated hemoglobin
ISPAD: International Society for Pediatric and Adolescent Diabetes
SD: standard deviation
SPSS: Statistical Package for the Social Sciences
T1DM: Type 1 diabetes mellitus
Y12HMC: Yekatit 12 Hospital Medical College

## Author Contributions

Mekashaw Wudu: conceptualization, data curation, formal analysis, investigation, methodology, and writing—original draft. Senbeto Gehano, Minase Shenkute, and Alemayehu Tsegaye: formal analysis, supervision, and writing—review and editing.

## Funding

No funding was received for this manuscript.

## Disclosure

All authors have read and approved the final version of the manuscript. Corresponding author had full access to all of the data in this study and takes complete responsibility for the integrity of the data and the accuracy of the data analysis.

## Ethics Statement

Ethical clearance was obtained from the Institutional Review Board (IRB) of Yekatit 12 Hospital Medical College (Approval Number: Y12HMCIRB/26/216/2023, dated July 12, 2023). An official permission letter was obtained from the Department of Pediatrics of Yekatit 12 Hospital Medical College prior to data collection. The study adhered to national research ethics guidelines and was conducted in accordance with the principles of the Declaration of Helsinki. Written informed consent was obtained from the parents or legal guardians of all participating children. Participation was entirely voluntary, and all participants had the right to withdraw from the study at any time without any consequences on their ongoing diabetic care and treatment. Confidentiality of all personal and medical information was rigorously maintained. Data were anonymized using unique participant codes, and access to information was strictly limited to authorized research personnel. All study procedures were conducted with respect and care for the best interests of the child participants.

## Conflicts of Interest

The authors declare no conflicts of interest.

## Supporting information


**Supporting Information** Additional supporting information can be found online in the Supporting Information section. 

## Data Availability

The data that support the findings of this study are available from the corresponding author upon reasonable request.

## References

[1] Djonou C. , Tankeu A. T. , Dehayem M. Y. , Tcheutchoua D. N. , Mbanya J. C. , and Sobngwi E. , Glycemic Control and Correlates in a Group of sub Saharan Type 1 Diabetes Adolescents, BMC Research Notes. (2019) 12, no. 1, 10.1186/s13104-019-4054-1, 30670077. PMC6341641 30670077

[2] Federal Ministry of Health Ethiopia , National Protocols for Noncommunicable Diseases Management, 2021, Federal Ministry of Health.

[3] Kidie A. A. , Ayal B. G. , Ayele T. , Fentie E. A. , and Lakew A. M. , Poor Glycemic Control and Associated Factors Among Pediatric Diabetes Mellitus Patients in Northwest Ethiopia, 2020: Facility-Based Cross Sectional Retrospective Study Design, Scientific Reports. (2022) 12, no. 1, 15664, 10.1038/s41598-022-19909-8, 36123389. PMC9485249 36123389

[4] Niba L. L. , Aulinger B. , Mbacham W. F. , and Parhofer K. G. , Predictors of Glucose Control in Children and Adolescents With Type 1 Diabetes: Results of a Cross-Sectional Study in Cameroon, BMC Research Notes. (2017) 10, no. 1, 10.1186/s13104-017-2534-8, 28606170. PMC5469010 28606170

[5] Katte J. C. , Agoons B. B. , Dimala C. A. , Bigna J. J. , and Sobngwi E. , Incidence and Prevalence of Type 1 Diabetes in Africa: A Systematic Review and Meta-Analysis Protocol, BMJ Open. (2022) 12, no. 9, 10.1136/bmjopen-2022-061605, 36109031. PMC9478842 36109031

[6] Shibeshi M. S. , Daba A. K. , Meiso K. M. , and Tadesse B. T. , Glycemic Control Among Children and Adolescents With Diabetes in Southern Ethiopia: A Cross-Sectional Study, BMC Endocrine Disorders. (2022) 22, no. 1, 10.1186/s12902-022-01070-y, 35705956. PMC9202171 35705956

[7] Noorani M. , Ramaiya K. , and Manji K. , Glycaemic Control in Type 1 Diabetes Mellitus Among Children and Adolescents in a Resource Limited Setting in Dar es Salaam, Tanzania, BMC Endocrine Disorders. (2016) 16, no. 1, 10.1186/s12902-016-0113-y, 27246505. PMC4886407 27246505

[8] Ngwiri T. , Were F. , Predieri B. , Ngugi P. , and Iughetti L. , Glycemic Control in Kenyan Children and Adolescents With Type 1 Diabetes Mellitus, International Journal of Endocrinology. (2015) 2015, 761759, 10.1155/2015/761759, 26494998. PMC4606130 26494998

[9] DiMeglio L. A. , Acerini C. L. , Codner E. , Craig M. E. , Hofer S. E. , Pillay K. , and Maahs D. M. , ISPAD Clinical Practice Consensus Guidelines 2018: Glycemic Control Targets and Glucose Monitoring for Children, Adolescents, and Young Adults With Diabetes, Pediatric Diabetes. (2018) 19, no. supplement 27, 105–114, 10.1111/pedi.12737, 30058221. 30058221

[10] Habteyohans B. D. , Gebremichael M. A. , Tesfaye D. M. , Woldemichael F. G. , Hailu A. D. , and Mekonnen T. K. , Poor Glycemic Control and Its Associated Factors Among children With Type 1 Diabetes Mellitus in Harar, Eastern Ethiopia: A Cross-Sectional study, BMC Endocrine Disorders. (2023) 23, no. 1, 10.1186/s12902-023-01453-9, 37759193. PMC10538014 37759193

[11] Huo L. , Yang J. , Liu Y. , Xu X. , Hu R. , and Weng J. , Factors Associated With Glycemic Control in Type 1 Diabetes Patients in China: a Cross-Sectional Study, Journal of Diabetes Investigation. (2020) 11, no. 6, 1575–1582, 10.1111/jdi.13282, 32323910. PMC7610125 32323910

[12] Al Zahrani A. M. and Al S. A. , Glycemic Control in Children and Youth With type 1 Diabetes Mellitus in Saudi Arabia, Clinical Medicine Insights: Endocrinology and Diabetes. (2019) 12, 1179551418825159, 10.1177/1179551418825159, 30718968. PMC6348502 30718968

[13] McLarty R. P. , Alloyce J. P. , Chitema G. G. , and Msuya L. J. , Glycemic Control, Associated Factors, Acute Complications of Type 1 Diabetes Mellitus in Children, Adolescents and Young Adults in Tanzania, Endocrinology, Diabetes & Metabolism. (2021) 4, no. 2, e00200, 10.1002/edm2.200, 33855206. PMC8029575 33855206

[14] Díez-Fernández A. , Ruiz-Grao M. C. , Mesas A. E. , Martínez-Vizcaíno V. , and Garrido-Miguel M. , Type 1 Diabetes Incidence Trends in children and adolescents Aged 0-14 Years in Europe: A Systematic Review and Meta-Analysis Protocol, BMJ Open. (2021) 11, no. 10, e054962, 10.1136/bmjopen-2021-054962, 34667016. PMC8527137 34667016

[15] Al-Qahtani S. M. , Abo-Shanab A. M. , Al-Shahrani A. M. , Alhazzani A. A. , Alqahtani M. S. , Asiri M. Y. , AlAsmari A. A. , Almahdi M. A. , Al Hassan A. A. , Alhassany A. M. , Shathan R. A. , Aldosari R. M. , AlQahtani A. S. , and Khalil S. N. , Factors Affecting Glycemic Control Among Saudi Children With Type 1 Diabetes Mellitus In Aseer Region, Southwestern Saudi Arabia, International Journal of Environmental Research and Public Health. (2022) 19, no. 18, 11558, 10.3390/ijerph191811558, 36141857. PMC9517271 36141857

[16] Dumrisilp T. , Supornsilchai V. , Wacharasindhu S. , Aroonparkmongkol S. , and Sahakitrungruang T. , Factors Associated With Glycemic Control in children and Adolescents With Type 1 Diabetes Mellitus at a Tertiary-Care Center in Thailand: A Retrospective Observational Study, Asian Biomedicine. (2017) 11, no. 6, 443–450, 10.1515/abm-2018-0021.

[17] Atwoli L. , Muhia J. , and Merali Z. , Ethiopian Pediatrics Society Guidelines, Ethiopian Journal of Pediatrics and Child Health. (2022) 17.

